# Prognostication of treatment non-compliance among patients with multidrug-resistant tuberculosis in the course of their follow-up: a logistic regression–based machine learning algorithm

**DOI:** 10.3389/fdgth.2023.1165222

**Published:** 2023-05-09

**Authors:** Denekew Tenaw Anley, Temesgen Yihunie Akalu, Anteneh Mengist Dessie, Rahel Mulatie Anteneh, Melkamu Aderajew Zemene, Wubet Alebachew Bayih, Yenealem Solomon, Natnael Atnafu Gebeyehu, Gizachew Ambaw Kassie, Misganaw Asmamaw Mengstie, Endeshaw Chekol Abebe, Mohammed Abdu Seid, Molalegn Mesele Gesese, Natnael Moges, Berihun Bantie, Sefineh Fenta Feleke, Tadesse Asmamaw Dejenie, Getachew Asmare Adella, Achenef Asmamaw Muche

**Affiliations:** ^1^ Department of Public Health, College of Health Sciences, Debre Tabor University, Debre Tabor, Ethiopia; ^2^ Department of Epidemiology and Biostatistics, Institute of Public Health, College of Medicine and Health Sciences, University of Gondar, Gondar, Ethiopia; ^3^ Faculty of Health Sciences, Curtin University, Perth, WA, Australia; ^4^ Geospital and Tuberculosis Research Team, Telethon Kids Institute, Perth, WA, Australia; ^5^ Department of Epidemiology and Preventive Medicine, Faculty of Medicine, School of Public Health and Preventive Medicine, Nursing and Health Sciences, Monash University, Melbourne, VIC, Australia; ^6^ Department of Maternal and Neonatal Health Nursing, College of Health Sciences, Debre Tabor University, Debre Tabor, Ethiopia; ^7^ Department of Medical Laboratory Science, College of Health Sciences, Debre Tabor University, Debre Tabor, Ethiopia; ^8^ Department of Midwifery, College of Medicine and Health Science, Wolaita Sodo University, Wolaita Sodo, Ethiopia; ^9^ Department of Epidemiology and Biostatistics, School of Public Health, Wolaita Sodo University, Wolaita Sodo, Ethiopia; ^10^ Department of Biochemistry, College of Health Sciences, Debre Tabor University, Debre Tabor, Ethiopia; ^11^ Unit of Physiology, Department of Biomedical Science, College of Health Science, Debre Tabor University, Debre Tabor, Ethiopia; ^12^ Department of Pediatrics and Child Health Nursing, College of Health Sciences, Debre Tabor University, Debre Tabor, Ethiopia; ^13^ Department of Comprehensive Nursing, College of Health Sciences, Debre Tabor University, Debre Tabor, Ethiopia; ^14^ Department of Public Health, College of Health Sciences, Woldia University, Woldia, Ethiopia; ^15^ Department of Medical Biochemistry, College of Medicine and Health Sciences, University of Gondar, Gondar, Ethiopia; ^16^ Department of Reproductive Health and Nutrition, School of Public Health, Wolaita Sodo University, Wolaita Sodo, Ethiopia; ^17^ HaSET Maternal and Child Health Research Program, Harvard T.H. Chan School of Public Health, Addis Ababa, Ethiopia; ^18^ Ethiopian Public Health Institute and Africa Research Excellence Fund, Addis Ababa, Ethiopia

**Keywords:** prediction, machine learning, treatment compliance, multidrug-resistant tuberculosis, Ethiopia

## Abstract

**Introduction:**

Drug compliance is the act of taking medication on schedule or taking medication as prescribed and obeying other medical instructions. It is the most crucial aspect in the treatment of chronic diseases particularly for patients with multidrug-resistant tuberculosis (MDR-TB). Drug non-compliance is the main reason for causing drug resistance and poor treatment outcomes. Hence, developing a risk prediction model by using early obtainable prognostic determinants of non-compliance is vital in averting the existing, unacceptably high level of poor treatment outcomes and reducing drug resistance among MDR-TB patients.

**Materials and methods:**

A retrospective follow-up study was conducted on a total of 517 MDR-TB patients in Northwest Ethiopia. A logistic regression–based machine learning algorithm was used to develop a risk score for the prediction of treatment non-compliance among MDR-TB patients in selected referral hospitals of Northwest Ethiopia. The data were incorporated in EpiData version 3.1 and exported to STATA version 16 and R version 4.0.5 software for analysis. A simplified risk prediction model was developed, and its performance was reported. It was also internally validated by using a bootstrapping method.

**Results:**

Educational status, registration group (previously treated/new), treatment support, model of care, and khat use were significant prognostic features of treatment non-compliance. The model has a discriminatory power of area under curve (AUC) = 0.79 with a 95% CI of 0.74–0.85 and a calibration test of *p*-value = 0.5. It was internally validated by using a bootstrapping method, and it has a relatively corrected discriminatory performance of AUC = 0.78 with a 95% CI of 0.73–0.86 and an optimism coefficient of 0.013.

**Conclusion:**

Educational status, registration group, treatment supporter, model of care, and khat use are important features that can predict treatment non-compliance of MDR-TB patients. The risk score developed has a satisfactory level of accuracy and good calibration. In addition, it is clinically interpretable and easy to use in clinical practice, because its features are easily ascertainable even at the initial stage of patient enrolment. Hence, it becomes important to reduce poor treatment outcomes and drug resistance.

## 1. Introduction

Multidrug-resistant tuberculosis (MDR-TB) is caused by a strain of mycobacterium tuberculosis (TB) resistant to at least two most potent TB drugs such as isoniazid and rifampicin (1). Anti-TB drug resistance is a public health issue of concern in both developing and industrialized nations. Although the incidence of TB has seen a decrease from the time when anti-TB drugs started becoming available, most low- and middle-income countries have been witnessing a revival of this illness (2). In 2017, 10 million cases of TB were reported worldwide. In the same year, 558,000 people developed rifampicin-resistant tuberculosis (RR-TB), of which 82% had MDR-TB (3). The global rates of incidence of MDR-TB are 3.5% and 18% in new and previously treated cases, respectively (3). According to a report by the World Health Organization (WHO), approximately 17% of global TB deaths result from MDR-TB (4).

The spread of drug-resistant TB is variable across different regions of the world. The rate of MDR-TB in China and India, the most populated countries of the world, accounts for 40% of all TB cases worldwide (5). Moreover, the high prevalence rates of MDR-TB among new cases are also a matter of serious concern in Estonia (14%), Latvia (9%), and Ivanova (9%), in the Russian Federation (6). However, the situation in low- and middle-income countries is highly variable. For instance, in Ethiopia, the prevalence rate of MDR-TB ranges from 0.5% to 46.3%, with a pooled rate of 7.24% (7).

Drug adherence/compliance is the most crucial step in drug therapy for chronic diseases, particularly for MDR-TB patients (8). The term “non-compliance” is frequently used to describe patients who disobey medical instructions (9). As far as therapeutic non-compliance is concerned, the consequences are not limited to its direct impacts such as treatment failure, as non-compliance has also been associated with negative impacts and external factors such as mortality, increased hospitalization and medical costs, and deteriorating levels of poverty in TB-affected households. Also, increased drug resistance will occur because of non-compliance (10). The rate of non-compliance is found to be different for medications that need to be taken for different periods. Thus, it is estimated that the compliance rate of long-term medication ranges between 40% and 50%. The rate of compliance for short-term therapy is much better, which ranges between 70% and 80% (11).

Shreds of evidence from previous studies revealed that patient-related factors such as educational status, attitude, patient–prescriber relationship, tobacco smoking, and alcohol intake influenced non-compliance. In addition, therapy-related factors such as route of administration, duration of the treatment period, medication side effects, and taste of the medication impacted non-compliance. Moreover, some factors related to the healthcare system such as a lack of accessibility, high travel expense, huge amount of time spent traveling to treatment centers, and a lack of family support all resulted in non-compliance (10–13). A study conducted in South Korea indicated that prediction of non-compliance is of great importance for achieving successful treatment outcomes for TB patients. It identified prognostic determinants such as younger age, lower body mass index (BMI), and history of TB for the development of a model for the prediction of non-compliance among TB patients (14).

However, to the best of our knowledge, there is no prior study on the development and validation of a risk score to predict non-compliance among MDR-TB patients in Northwest Ethiopia. Therefore, developing risk prediction model by using early obtainable prognostic determinants of non-compliance is vital in averting the existing, unacceptably high level of poor treatment outcomes among MDR-TB patients. To make clear what this study aims for, three important points are to be noted: the first is to determine the rate of treatment non-compliance, the second is to develop a risk prediction score for treatment non-compliance, and the last is to internally validate the developed model. Given these aims, it is hoped that the findings from this study will help healthcare providers identify clients who are at a high risk of non-compliance and provide the necessary intervention before unwanted health outcomes and increased drug resistance occur.

## 2. Materials and methods

### 2.1. Study design and setting

A multicenter retrospective follow-up study design was employed in two treatment-initiating centers (TICs) in Northwest Ethiopia in the period between September 2010 and July 2020. The period of data collection was between 21 January 2021 and 7 July 2021. The University of Gondar Compressive Specialized Hospital (UoGCSH) was the first TIC, which is located 737 km from Ethiopia’s capital city, Addis Ababa. The hospital is one of the region’s largest tertiary-level teaching and referral facilities. Debre Markos Referral Hospital, located 300 km from Addis Ababa, was the second TIC.

### 2.2. Population

The source population consisted of all MDR-TB patients enrolled in Northwest Ethiopia, while the study group consisted of those enrolled in the two TICs. All MDR-TB patients who underwent follow-ups at the UoGCSH and Debre Markos Referral Hospital were included in the study.

### 2.3. Eligibility

All MDR-TB patients who underwent follow-ups in the UoGCSH and Debre Markos Referral Hospital in the last 10-year period were included in the study. Patients for whom outcomes were not ascertained were excluded.

### 2.4. Variables of the study

Treatment non-compliance (non-adherence) was the outcome variable. Patients who missed ≥10% of the total prescribed dose were considered by clinicians as non-adherent (15, 16). Furthermore, patients who did not obey any of the medical instructions were labeled as non-compliants. This ascertainment was made on the basis of patients’ responses and/or clinicians’ observations. The prognostic determinants were sex, age, residence, educational status, treatment supporter, registration group, functional status at admission, regimen type, comorbidity, baseline anemia, alcohol use, BMI, major adverse event, and khat use. The term “khat” refers to the leaves and young shoots of *Catha edulis*. The plant has been widely used since the thirteenth century as a recreational drug by the indigenous people of East Africa, the Arabian Peninsula, and the Middle East. At the baseline assessment in this study, patients were labeled as khat users if they were found to be the actual users of this substance. Patients who had someone with them who could help them financially and/or assist them in their treatment were described as those having treatment support. In addition, patients who had been previously treated for MDR-TB were described as previously treated in the registration-type category. With regard to functional status, patients who were seriously ill and unable to walk alone were labeled as bedridden and otherwise as ambulatory. The above-mentioned independent variables are easily ascertainable at the time of the patients’ enrolment and identified from different literatures mentioned in the introduction section of this study. Quantitative variables were categorized for the sake of simplicity and easy applicability of the risk score developed.

### 2.5. Study size

A detailed description and determination of the study size were written and published elsewhere (17).

### 2.6. Data collection procedure and quality control

A structured data-extracting tool (checklist) was designed using various literatures (17–28) and pieces of evidence found in the patients’ medical reports. Sociodemographic characteristics, treatment-related factors, comorbidities, and behavioral aspects were retrieved as prognostic drivers of treatment non-compliance. Body mass index was computed by dividing their baseline weight in kilograms by their height in meters squared. A pretest was performed in 5% of the sample size to check how comprehensive the checklist was and to identify any inconsistencies and discrepancies. Data collectors were given 2 days of training on the data collection procedure and techniques. The collected data were double-checked for completeness and accuracy on a daily basis.

### 2.7. Data processing and analysis

Epi Info version 7 software was used to incorporate the coded data comprising prognostic determinants. They were then analyzed using STATA version 16 and R version 4.0.5 statistical software. The “mice” package in R was used to compensate for the missing data by employing a variety of imputation strategies under the assumption that the data were missed at random (MAR). Five imputed datasets were created when the imputation process was completed on the entire dataset. The details of the imputation process are published elsewhere (17). For this particular study, the features used for the model development were found to be complete, except those for the educational status variable (missed for 2.9% of the observations).

For categorical variables, descriptive statistics, frequencies, and percentages were used. The Kolmogorov–Smirnov test was used to determine the normality of distribution. Mean and standard deviation were used to summarize normally distributed continuous variables. Median and interquartile ranges (IQRs) were used to describe variables where the normality assumption failed.

### 2.8. Model development and validation

The binomial logistic regression model was used to develop the risk prediction model to predict treatment non-compliance. Univariable analysis was performed to select the prognostic determinants of non-compliance. A forward stepwise selection method was used to develop a more simplified model with a reduced risk of overfitting. Variables with a *p*-value of 0.25 and less in univariable analysis were incorporated in the multivariable analysis. A statistically significant association was declared when the *p*-value was 0.05 and less. The risk prediction score was created from the final simplified multivariable logistic regression model.

The prediction ability of the generated risk score was assessed in terms of discriminatory power and calibration. The discriminatory strength of the resulting risk score was measured using *C*-statistics in receiving operating characteristic (ROC) curve analysis. The *C*-statistics could range from 0.5 (no predictive ability) to 1 (perfect discrimination) (24, 25). To visualize the model’s calibration performance, the calibration plot and Hosmer–Lemeshow test were used.

The calibration of the model was presented graphically by using the calibration plot and Hosmer–Lemeshow test. The developed model was also evaluated in terms of accuracy and misclassification rate. Furthermore, we performed a bootstrap resampling of the original dataset with 10,000 repetitions for internal validation to calculate relatively corrected *C*-statistics. In addition, the net benefit of the model in clinical practice was assessed by using decision curve analysis (DCA) metrics.

The study was reported in accordance with the TRIPOD (transparent reporting of a multivariable prediction model for individual prognosis or diagnosis) statement.

### 2.9. Risk score development

The risk score was calculated by dividing each coefficient by the lowest beta coefficient. Then, we determined the total score for each patient by assigning the points for each variable present and adding them up. Treatment non-compliance was grouped as low, intermediate, and high risk. For the sake of classifying patients as low and high risk of treatment non-compliance, the score was transformed into a dichotomous prediction test. Finally, the performance of the risk score in terms of sensitivity and specificity was assessed for different thresholds of 4, 5, 6, and 7.

## 3. Results

### 3.1. Sociodemographic characteristics of the study subjects

A total of 517 patients were involved in the study, and the flow diagram of the participant selection is published elsewhere (17). Of the total samples, 321 (62.1%) participants were males. The median age of the participants was found to be 30 years with an IQR of 17 years (Table 1).

**Table 1:** Sociodemographic and behavioral characteristics of MDR-TB patients.

Characteristics	Frequency (%)
Gender	
Male	321 (62.1)
Female	196 (37.9)
Age (median ± IQR)	30 ± 17
Occupation	
Government employee	33 (6.4)
Self-employed	69 (13.4)
Farmer	217 (42)
Unemployed	8 (1.5)
Student	75 (14.5)
Daily laborer	92 (17.8)
Others*	23 (4.4)
Educational status	
No formal education	193 (37.4)
Primary	192 (37.1)
Secondary	90 (17.4)
Tertiary	42 (8.1)
Treatment supporter	
Yes	435 (84.1)
No	82 (15.9)
Baseline smoking status	
No	493 (95.4)
Yes	24 (4.6)
Baseline alcohol use	
No	373 (72.1)
Yes	144 (27.9)
Baseline khat use	
No	499 (96.5)
Yes	18 (3.5)

MDR-TB, multidrug-resistant tuberculosis; IQR, interquartile range.

* Represents young children/preschool children.

### 3.2. Clinical characteristics of the study subjects

Approximately 81.6% of the study subjects had a history of anti-TB treatment. Approximately 47.9% of the patients were found to be anemic, and most of them (83.2%) suffered from radiological abnormalities (Table 2).

**Table 2:** Clinical characteristics of MDR-TB patients in Northwest Ethiopia.

Characteristics	Frequency (%)
Type of drug regimen	
Long-term regimen	475 (91.9)
Short-term regimen	42 (8.1)
Registration group	
Previously treated	422 (81.6)
New	95 (18.4)
Model of care	
Hospitalized	483 (93.4)
Ambulatory	34 (6.6)
Site of the disease	
Pulmonary	461 (89.2)
Extrapulmonary	56 (10.8)
Comorbidity	
No	345 (66.7)
Yes	172 (33.3)
HIV co-infection	
No	83 (74.1)
Yes	134 (25.9)
Major adverse event	
No	446 (86.3)
Yes	71 (13.7)
Complications	
No	390 (75.4)
Yes	127 (24.6)
Type of resistance	
Mono	315 (60.9)
MDR	163 (31.5)
Poly	39 (7.5)
Baseline culture	
Positive	368 (71.2)
Negative	149 (28.8)
Baseline sputum smear	
Positive	316 (61.1)
Negative	201 (38.9)
Baseline anemia	
No	270 (52.2)
Yes	247 (47.8)
Radiological abnormalities	
Cavitary lesions	139 (26.9)
Non-cavitary lesions	291 (56.3)
Normal	87 (16.8)
Body mass index (kg/m^2^)	
<18.5	367 (71.0)
≥18.5	150 (29.0)

MDR-TB, multidrug-resistant tuberculosis; HIV, human immunodeficiency virus.

### 3.3. Treatment non-compliance

**Figure 1:**
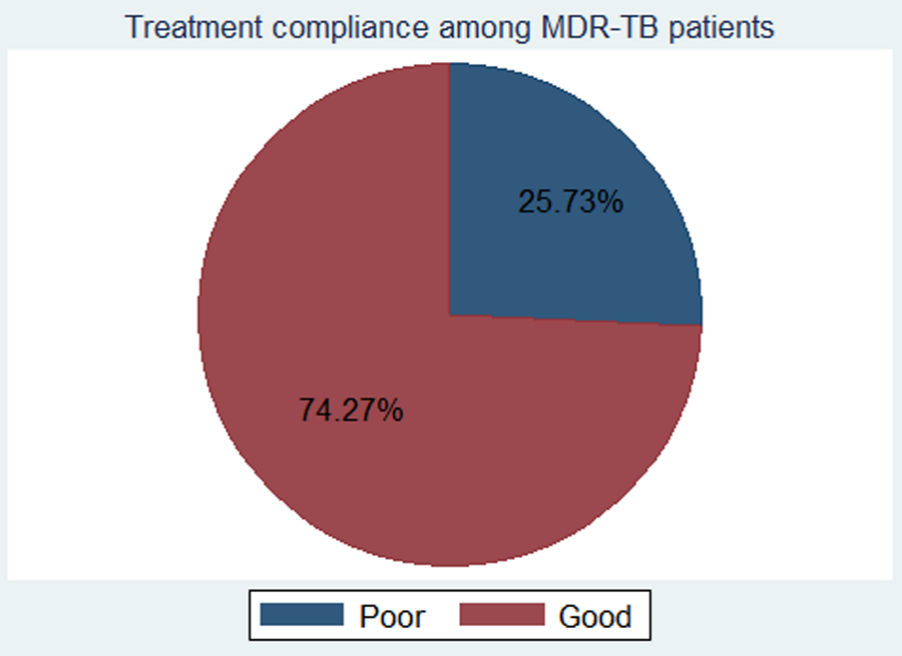
The rate of treatment non-compliance among MDR-TB patients in Northwest Ethiopia, from September 2010 to July 2020. MDR-TB, multidrug-resistant tuberculosis.

The rate of treatment non-compliance among the study subjects was found to be 25.7% with a 95% CI of 22.1–29.7 (Figure 1).

There was a difference in the treatment outcome of the patients with respect to their status of non-compliance [Pearson χ^2^(1) = 252.40, Pr = 0.000]. A majority of the patients with treatment non-compliance ended up with poor treatment outcomes (84.96%).

### 3.4. Prediction model development

A total of 14 features were considered potential prognostic determinants of treatment non-compliance among MDR-TB patients. Eight of them were incorporated in the multivariable analysis model. These were sex, age, educational status, occupational status, registration group, model of care, treatment supporter, and khat use. The prediction model was developed, and its equation was obtained using five predictors identified as significant in the multivariable analysis. A simplified risk score was computed by dividing the coefficients by the smallest coefficient and rounding them to the nearest integer (Table 3).

**Table 3:** Coefficients and risk scores of predictors incorporated in the model to predict treatment non-compliance in MDR-TB patients.

Predictors	Univariable analysis		Multivariable analysis		Simplified risk score
	Coef. (95% CI)	*p*-value	Coef. (95% CI)	*p*-value	
Gender					
Female	Reference				
Male	−0.11 (−0.62 to 0.39)	0.102	−0.13 (2.01 to 0.12)	0.651	—
Age (years)					
≤45	Reference				
45 and above	0.41 (−0.32 to 1.15)	0.165	−0.81 (−1.8 to 0.18)	0.753	—
Educational status					
Literate	Reference				
Illiterate	0.84 (0.27 to 1.40)	0.003	1.17 (0.44 to 1.91)	0.007	2
Occupational					
Government employed	Reference				
Farmer	0.35 (−0.34 to 1.04)	0.23	0.15 (−1.21 to 1.25)	0.543	
Unemployed	0.32 (−0.26 to 0.91)	0.19	0.23 (−0.25 to 1.05)	0.971	—
Registration group					
New	Reference	0.023	0.65 (0.45 to 0.82)	0.013	1
Previously treated	0.67 (0.51 to 0.79)				
Treatment supporter					
Yes	Reference				
No	1.1 (0.49 to 1.637)	<0.001	0.58 (0.10 to 1.26)	0.011	1
Model of care					
Hospitalized	Reference				
Ambulatory	1.3 (0.48 to 2.04)	0.012	1.27 (0.40 to 2.14)	0.006	2
Khat use					
No	Reference				
Yes	2.2 (1.57 to 2.79)	<0.001	2.18 (1.49 to 2.85)	<0.001	4
Intercept	−3.56 (−4.77 to −2.54)	<0.001			

MDR-TB, multidrug-resistant tuberculosis; CI, confidence interval.

### 3.5. Performance of the model with original beta coefficients

**Figure 2:**
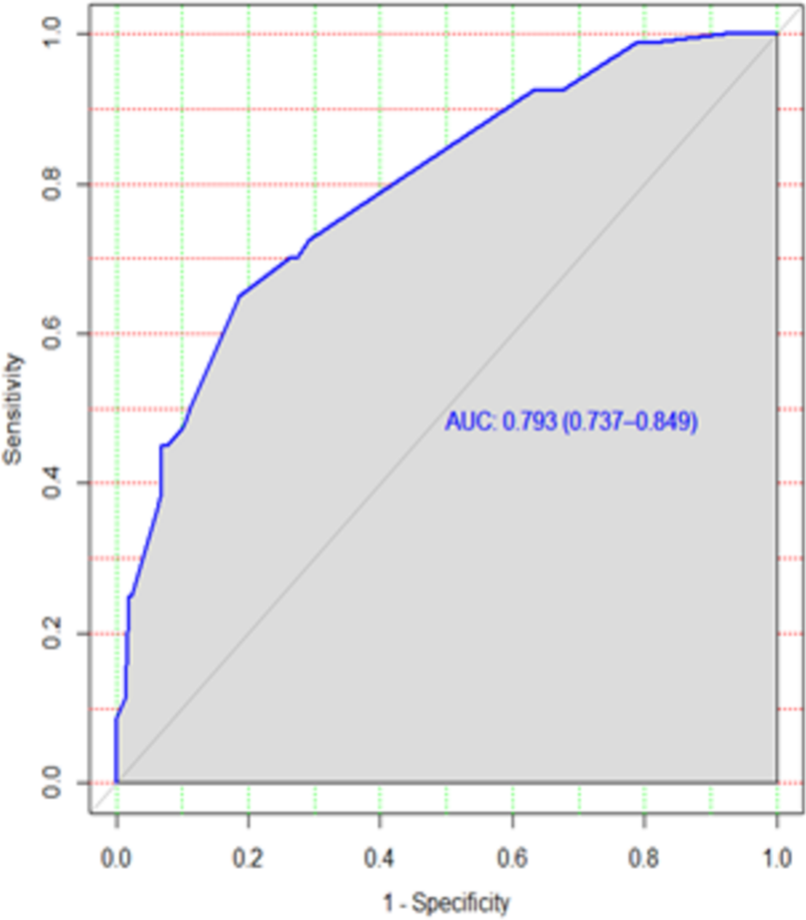
ROC curve of the risk prediction score for the prediction of treatment non-compliance among MDR-TB patients in Northwest Ethiopia. ROC, receiving operating characteristic; MDR-TB, multidrug-resistant tuberculosis.

The discriminatory power of the model with original beta coefficients was found to have an area under curve (AUC) of 0.793 with a 95% CI of 0.736–0.848 (Figure 2). The estimated risk of treatment non-compliance = 1/(1 + exp − (−3.56 + 1.17  ×  educational status (illiterate) + 0.65 × registration group (previously treated) + 0.58 × treatment supporter(no) + 1.27 × model of care (ambulatory) + 2.18 × khat use (yes).

**Figure 3:**
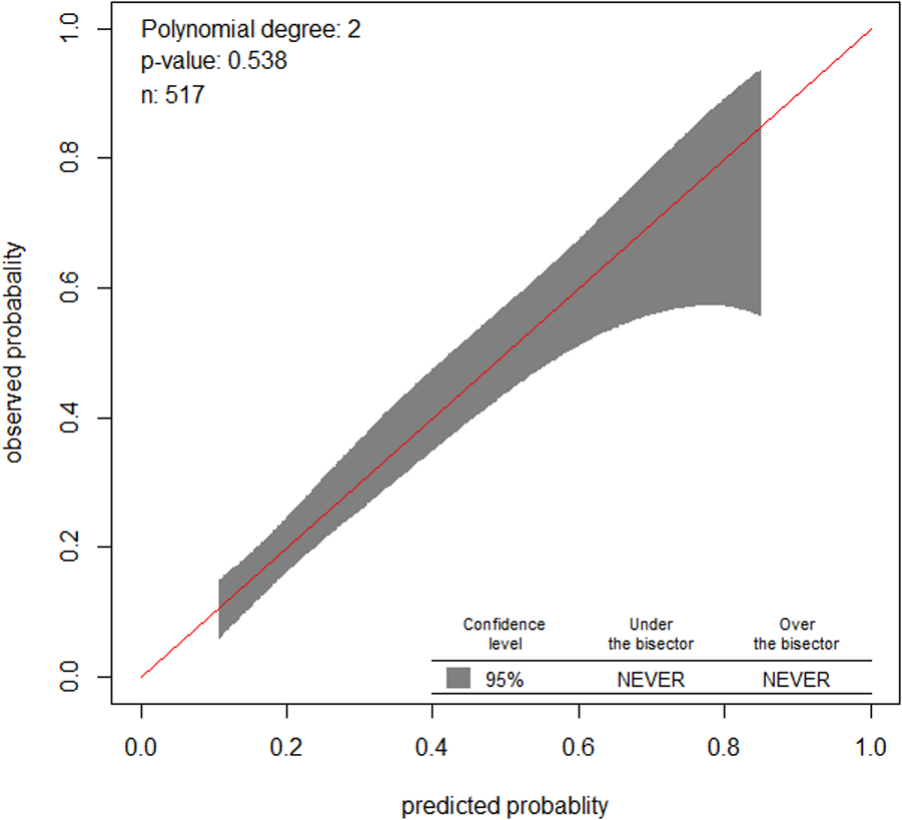
Observed vs. predicted probabilities of treatment non-compliance in a sample.

Similarly, the calibration test of the model had a *p*-value of 0.5, which meant that the model did represent the data well (Figure 3). The prediction ability of individual significant prognostic determinants was assessed, and the history of treatment was found to have the highest predictive ability of AUC = 0.62.

A bootstrapping technique with 10,000 repetitions was used to test the model in terms of validity. This method was preferred over other methods of validation, because the sample size is relatively small. The intercept, original coefficients of each feature, the bias, and standard error were identified by the bootstrapping technique. The optimism coefficient was found to be only 0.061, and this low optimism coefficient indicated that the model was less likely to be sample dependent.

### 3.6. Performance of the simplified risk score

**Figure 4:**
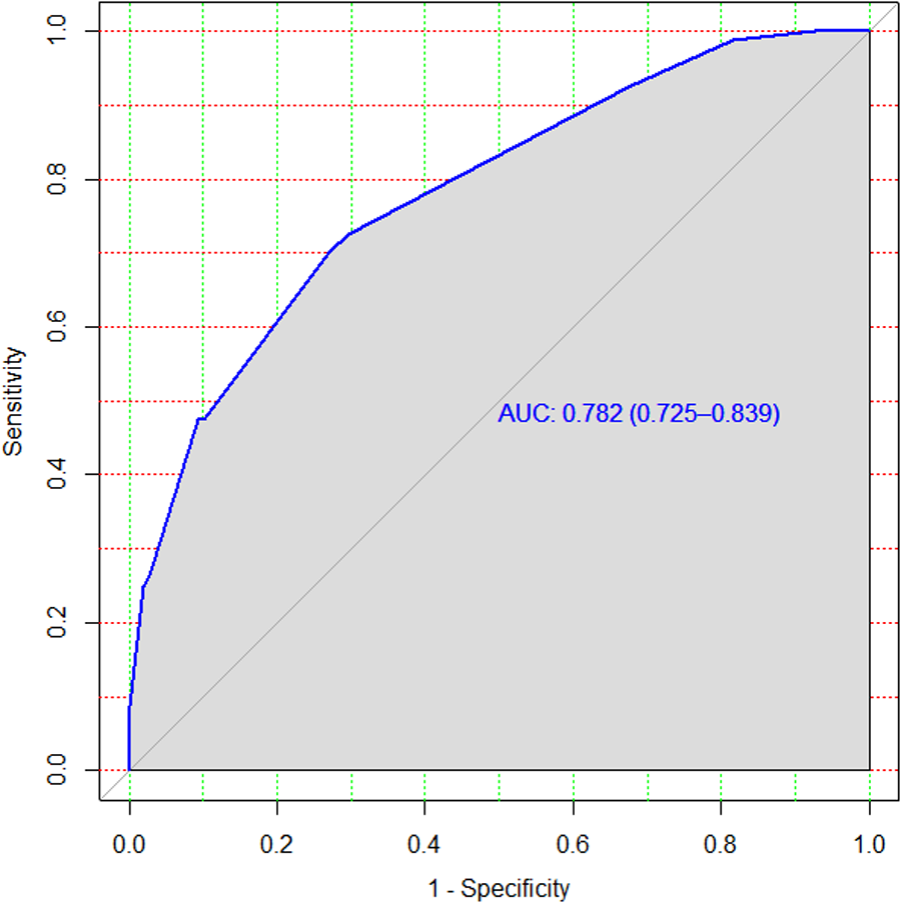
ROC curve of the model after internal validation using the bootstrapping method.

The rounding of regression coefficients in the final model offered a simplified risk score of treatment non-compliance. The risk score displayed almost similar discriminatory performance with original beta coefficients, with an AUC of 0.782 and a 95% CI of 0.73–0.83 (Figure 4). However, the model incorrectly predicted (misclassified) the outcome for 18% of the patients with an accuracy rate of 82%.

### 3.7. Risk classification using a simplified risk score

The minimum and maximum scores that a patient can have are 0 and 10, respectively. The incidence of the outcome in low-risk (<4), intermediate-risk (4–7), and high-risk groups (≥8) were 8.1%, 30.98%, and 54.4%, respectively (Table 4).

**Table 4:** Risk classification of MDR-TB patients for treatment non-compliance.

Score^a^ (risk category)	Number of patients (%)	Incidence of treatment delay
Low risk (<4)	248 (47.97%)	20 (8.06%)
Intermediate (4–7)	155 (29.98%)	48 (30.98%)
High risk (≥8)	114 (22.05%)	62 (54.4%)
Total	517 (100%)	130 (25.7%)

MDR-TB, multidrug-resistant tuberculosis.

^a^ A score developed by the model.

The risk score of treatment non-compliance = 2 × educational status (no formal education) + 1 × registration group (previously treated) + 1 × treatment supporter (no) + 2 × model of care (ambulatory) + 4 × khat use (yes).

The optimum cutoff point for the risk score was determined by the Youden index method. Patients having a risk score of <4 were classified as low risk, and high risk was determined when the patients had a risk score of ≥4. In addition, the Youden index value of the predicted probability was found to be 0.32, indicating that patients with a risk probability of ≥0.32 would be classified as those at a higher risk of treatment non-compliance. Table 5 summarizes the performance of the risk score at different thresholds.

**Table 5:** Performance of the risk score at different thresholds.

Thresholds^a^	Sensitivity	Specificity	PPV	NPV
>4	72.5%	70.3%	44.3%	88.9%
>5	65%	81.5%	53.1%	87.8%
>6	47.5%	89.9%	60.3%	84.2%
>7	41.25%	93.1%	66%	82.9%

PPV, positive predictive value; NPV, negative predictive value.

^a^ Risk scores.

### 3.8. Decision curve analysis

Decision curve analysis is a straightforward, innovative technique for assessing predictive models in terms of public health and clinical utility. The thin black line represents the assumption that all patients are at risk of treatment non-compliance, while the thick black line reflects the assumption that none of the patients are at risk of treatment non-compliance. The red line shows the developed prediction model.

The conventional net benefit of employing either the model or the two extreme methods (all or none approaches) is shown on the *y*-axis of the curve, while various threshold probabilities with potential cost–benefit ratios are shown on the *x*-axis.

**Figure 5:**
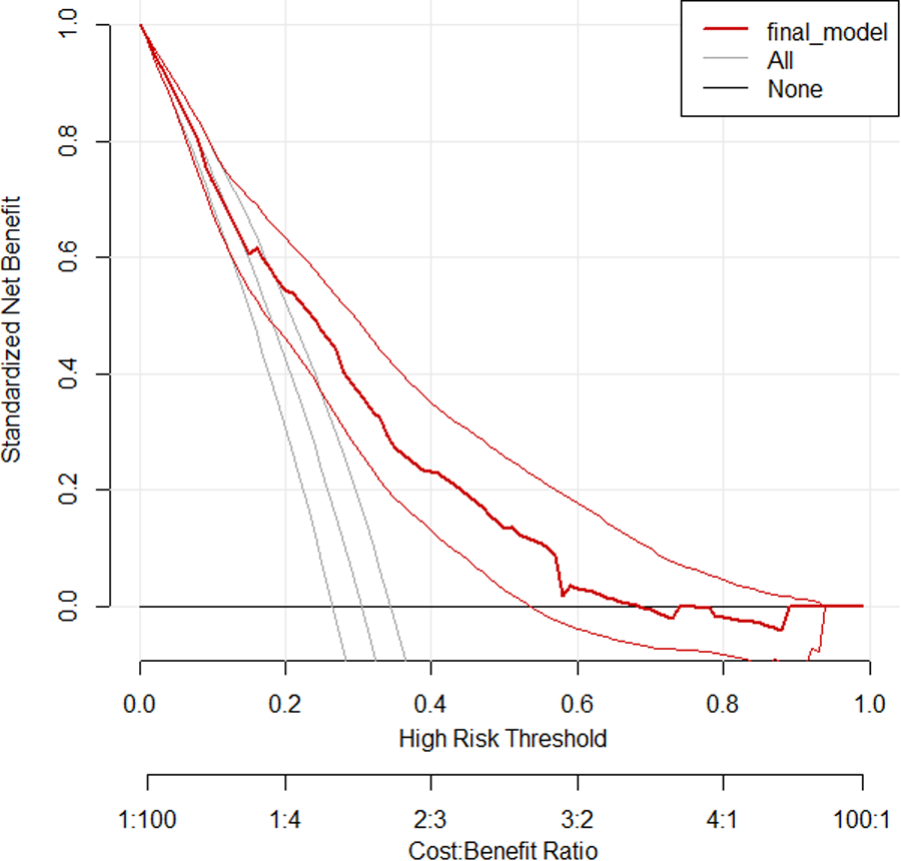
Decision Curve Analysis illustrating the net benefit of the developed model.

Therefore, across a range of threshold probabilities, the curve generally illustrates the standard net benefit of employing the model, which is in contrast to the other two approaches. As shown in the graph below, the model has higher net benefits for a majority of threshold probabilities compared with the two approaches described above. The model has no role for the prediction of the treatment non-compliance for threshold probabilities <0.18 and >0.64 (Figure 5).

## 4. Discussion

Treatment non-compliance is associated with poor treatment outcomes and even furthers drug resistance in TB (7, 18, 23). In low-income countries such as Ethiopia, which is beset with a myriad problems, treatment non-compliance continues to prevail and increases the risk of drug resistance (18, 23). The magnitude of treatment non-compliance identified in this study is higher than that found in a study conducted in Southern Ethiopia (24.5%) and the Amhara region (14.5%) (15, 19).

The patient-specific model of care is usually recommended by many researchers because the level of treatment adherence or compliance differs from patient to patient. This is also regarded as the main factor for the development of drug resistance in the treatment and care of TB across different areas in the developing regions of the world. Hence, in this study, we developed an individualized risk prediction model by using prognostic determinants (mainly sociodemographic and behavioral factors) that we obtained from different literatures, which are reported as factors influencing treatment compliance. These variables were educational status, registration group, treatment support, model of care, and khat use. The educational status of the patients was one of the important prognostic determinants of treatment non-compliance among MDR-TB patients. A study conducted in Sudan identified the education level of the patients as the main factor impacting treatment non-compliance (26). A lack of knowledge about the severity of the illness, its potential for spread, and its potential for fatality have an impact on patients’ adherence to treatment, emphasizing the necessity of providing all patients with adequate health information commencing at the point of their enrollment.

Treatment support was also an important feature in predicting treatment non-compliance. Social support in terms of money and companionship was of great importance for the treatment adherence and outcome of MDR-TB patients. A lack of money caused people to go without meals and pay for transportation, which made it difficult for TB patients to continue receiving treatment. This finding was in line with a systematic review and meta-analysis conducted in China, Brazil, and Myanmar and a systematic review conducted in developing countries (19, 23, 29–31).

Patients with a history of anti-TB treatment were found to have a higher risk of treatment non-compliance. This could be attributed to their reduced trust in the effectiveness of the treatment because of the failure of the previous treatment. Moreover, the burden of medication to which they were exposed and the second phase of long treatment might compel them to give up early, unless necessary measures are taken by the health professionals delivering the care. Hence, a good patient–physician relationship is necessary to deepen patients’ commitment to accept and adhere to the long period of treatment and the high pill burden that they are likely to face (23).

With regard to the model of care, patients in an ambulatory model of care may have a problem with medication adherence because they may not undergo a strict follow-up regimen and enjoy good companionship with health professionals, unlike their counterparts in the hospitalized model of care. This finding is in line with the study conducted in Addis Ababa (32).

The use of illicit drugs was also a significant predictor of treatment non-compliance. This was because patients who consumed illicit drugs such as khat might not be in a position to provide a commitment to adhere to the anti-MDR-TB treatment. Similar to our study findings, studies conducted in Iran and other developing countries identified smoking and illicit drug use as predictors of treatment non-compliance (23).

After we realized that treatment compliance was the key to improve treatment outcomes for MDR-TB patients and prevent drug resistance, we decided to prepare an individualized risk prediction score model using the easily ascertainable prognostic determinants discussed above. This model was prepared for the whole dataset containing 517 samples and tested using the bootstrapping method. Its performance was assessed using appropriate performance metrics such as discrimination and calibration. The model had nearly 80% discriminatory power. This indicated that the model had a satisfactory level of discrimination accuracy. This performance was comparable with that of other prediction models designed in Peru (AUC, 75.5%) (33) and South Korea (AUC, 79%) on treatment failure and compliance in patients with TB (14). The prediction accuracy of the risk score was better than that in a study conducted on the prediction of lost to follow-up (*C*-index = 0.65) and death (*C*-index = 0.70) among MDR-TB patients and the prediction model of poor treatment outcomes among MDR-TB patients (*C*-statistics = 0.69) (28, 34, 35). However, this model had a lower prediction accuracy compared with that in a study conducted in China on individualized predictions of incident MDR-TB after the completion of pulmonary TB treatment (*C*-index = 0.83) (36). This could be due to a difference in terms of the quality of data recording and handling. This difference could be attributed to the sample size.

The calibration of the model was assessed in both the Hosmer–Lemeshow test and the calibration plot, which showed that the model well represented the data. In addition, the model was internally validated and was found to have a small optimism coefficient of 0.013. This finding indicated that the model was less likely to be overfitted. Hence, the model could be a robust one, less affected by sample difference, in turn, indicating its transferability.

Based on the identified optimum cutoff point (4), we classified patients into three risk levels. These were low, intermediate, and high risks. The minimum and maximum scores that a patient could have were 0 and 10, respectively. The rates of incidence of the outcome in the low-risk (<4), intermediate-risk (4–7), and high-risk groups (≥8) were 8.1%, 30.98%, and 54.4%, respectively. This showed that the highest rate of incidence of non-compliance was found from patients classified as having a higher risk of the outcome; this, in turn, indicated the strength of the built model. In addition, the DCA identified the model as a clinically interpretable one with a higher net benefit of using it in the management and care of MDR-TB patients. It was found to be better than the “treat all or none” approach, as evidenced by the decision curve, in which the model showed a higher standard net benefit for a majority of threshold probabilities (0.18–0.64). Offering interventions on the basis of the status of the clinical setting and the skill level of care providers is therefore of great importance in the management of MDR-TB patients for the higher-risk groups identified by the model. Generally, the developed simplified risk score for the prediction of treatment non-compliance among MDR-TB patients is easier to use in a routine clinical and public health practice, because it is constructed using early and easily ascertainable prognostic determinants. To the best of our knowledge, this is the first of its kind treatment non-compliance risk prediction score model developed in a country like Ethiopia. The model’s strengths are that it has satisfactory prediction performance and it is also not confined to a single site, hence enhancing its applicability in external settings. In addition, the identified small optimism coefficient shows that the model is less likely to be sample dependent. However, the model is based on retrospective data, which might cause limitations in terms of data completeness and availability of important predictors for an accurate prediction of treatment non-compliance in patients with MDR-TB.

## 5. Conclusion

Educational status, registration group, treatment support, model of care, and khat use are important features that can predict treatment non-compliance in the course of treating MDR-TB patients. The risk score developed in this study has a satisfactory level of accuracy and good calibration. In addition, it is clinically interpretable and easy to use in clinical practice, because its features are easily ascertainable even at the initial stage of patient enrollment. Hence, it is important to reduce poor treatment outcomes and drug resistance.

## Data Availability

The raw data supporting the conclusions of this article will be made available by the authors, without undue reservation.
